# EHMTI-0165. Acetazolamide infusion induces immediate and delayed headache and intracranial artery dilatation in healthy volunteers

**DOI:** 10.1186/1129-2377-15-S1-E2

**Published:** 2014-09-18

**Authors:** N Arngrim, HW Schytz, MS Asghar, FM Amin, A Hougaard, VA Larsen, PJH de Koning, HBW Larsson, J Olesen, M Ashina

**Affiliations:** 1 Danish Headache Center and Department of Neurology, Glostrup Hospital Faculty of Health and Medical Sciences Copenhagen University, Glostrup, Denmark; 2 Department of Radiology, Rigshospitalet Faculty of Health and Medical Sciences Copenhagen University, Copenhagen, Denmark; 3 Division of Image Processing Department of Radiology, Leiden University Medical Center, Leiden, Denmark; 4 Functional Imaging Unit Diagnostic Department, Glostrup Hospital Faculty of Health and Medical Sciences Copenhagen University, Glostrup, Denmark

## Background

All headache provoking substances are vasoactive and induce an immediate headache in healthy volunteers. The carbonic anhydrase inhibitor acetazolamide causes dilatation of cerebral arterioles and extracellular acidosis. Whether acetazolamide induces headache and dilatation of cranial arteries is unknown.

## Aim

To test whether acetazolamide induces headache and dilatation of cranial arteries in healthy volunteers.

## Methods

Twelve healthy women received injection of 1 g acetazolamide or placebo on two separate days in a randomized double-blind crossover study design. We recorded headache on a verbal rating scale from 0-10 during an immediate phase (0-90 min) and a delayed phase (2-12 h). The circumferences of cranial arteries were measured in a blinded fashion using 3T high-resolution magnetic resonance angiography 30 and 60 min after acetazolamide injection.

## Results

Acetazolamide induced immediate (0-30 min) headache in 9 out of 12 participants compared to 3 out of 12 participants after placebo (P =0.031). 11 out of 12 participants reported headache in the delayed phase (2-12 h) after acetazolamide compared to 4 out of 12 after placebo (P = 0.016). Arterial circumference of the intracranial arteries increased after acetazolamide compared to placebo (P ≤ 0.005).

## Conclusion

In contrast to known headache provoking substances, acetazolamide induced not only immediate but also a delayed headache. In addition, acetazolamide induced dilatation of intracranial arteries. It is possible that extracellular acidosis induced by acetazolamide causes sensitization of intracranial perivascular nociceptors, which, in combination with vasodilatation, leads to delayed headache in healthy volunteers.

No conflict of interest.

